# Supplementary material for: Chemotaxis response and age-stage, two-sex life table of the Cheilomenes sexmaculata (Fabricius) (Coccinellidae: Coleoptera) against different aphid species
Source: PLoS One. 2024 Feb 2;19(2):e0289682. doi: 10.1371/journal.pone.0289682 (PMC10836708; doi:10.1371/journal.pone.0289682)

PLOS ONE

Chemotaxis response and age-stage, two-sex life table of the Cheilomenes sexmaculata (Fabricius) (coccinellidae: coleoptera) against different aphid species

--Manuscript Draft--

**Manuscript Number:**	PONE-D-22-16646
**Article Type:**	Research Article
**Full Title:**	Chemotaxis response and age-stage, two-sex life table of the Cheilomenes sexmaculata (Fabricius) (coccinellidae: coleoptera) against different aphid species
**Short Title:**	Chemical behavioral response of Cheilomenes sexmaculata against different aphid species
**Corresponding Author:**	Syed Muhammad Zaka<br>Bahauddin Zakariya University Faculty of Agriculture Science and Technology<br>Multan, PAKISTAN
**Keywords:**	Zigzag beetle; Diuraphis noxia; Toxoptera citricida; Lipaphis erysimi; life table; olfaction
**Abstract:**	The Cheilomenes sexmaculata (Fabricius) (Coleoptera: Coccinellidae), is one of the most beneficial and identifiable predators of numerous soft-bodied and sucking insect pests of several crops. Biological parameters and olfactory response of C. sexmaculata were investigated under laboratory conditions by providing three different aphid species i.e., mustard aphid (Lipaphis erysimi), citrus black aphid (Toxoptera citricida), and peach aphid (Diuraphis noxia) as a food source. The developmental period of immature stages of C. sexmaculata was shorter on D. noxia as compared to other aphid species. The adult longevities were longer on D. noxia and T. citricida while shorter on L. erysimi . Female fecundity was highest on D. noxia while lowest on L. erysimi . Life table parameters i.e., intrinsic rate of increase (r), finite rate of increase (λ), net reproductive rate (R o), and gross reproductive rate (GRR) were maximum on D . noxia while minimum on L. erysimi. The mean generation time C. sexmaculata was 20.90, 23.69, and 26.2 days on D. noxia , L. erysimi , T . and citricida,, respectively. These findings were further confirmed from the olfactory experiment where D. noxia proved to be the most preferred prey. This study provides necessary information for mass-rearing of C. sexmaculata .
**Order of Authors:**	Hafiz Muhammad Safeer, M.Phil
	Aimen Ishfaq, M.Phil
	Syed Muhammad Zaka
	Alia Tajdar, M.Phil
	Ahmad Saood, Bachelors
	Zuraiz Ali Shah, Bachelors
	Muhammad Shahzaib, M.Phil
	Khalid Abbas, M.Phil
	Adeel Mukhtar, M.Phil
	Muhammad Usama Altaf, M.Phil
**Additional Information:**	
**Question**	**Response**
**Financial Disclosure**<br><br>Enter a financial disclosure statement that describes the sources of funding for the work included in this submission. Review	The author(s) received no specific funding for this work

Powered by Editorial Manager® and ProduXion Manager® from Aries Systems Corporation

the submission guidelines for detailed requirements. View published research articles from *PLOS ONE* for specific examples.<br><br>This statement is required for submission and **will appear in the published article** if the submission is accepted. Please make sure it is accurate.<br><br>**Unfunded studies**<br>Enter: *The author(s) received no specific funding for this work.*<br><br>**Funded studies**<br>Enter a statement with the following details:<br>• Initials of the authors who received each award<br>• Grant numbers awarded to each author<br>• The full name of each funder<br>• URL of each funder website<br>• Did the sponsors or funders play any role in the study design, data collection and analysis, decision to publish, or preparation of the manuscript?<br>• **NO** - Include this sentence at the end of your statement: *The funders had no role in study design, data collection and analysis, decision to publish, or preparation of the manuscript.*<br>• **YES** - Specify the role(s) played.<br><br>* typeset	
**Competing Interests**<br><br>Use the instructions below to enter a competing interest statement for this submission. On behalf of all authors, disclose any competing interests that could be perceived to bias this work—acknowledging all financial support and any other relevant financial or non-financial competing interests.<br><br>This statement is required for submission and **will appear in the published article** if the submission is accepted. Please make sure it is accurate and that any funding sources listed in your Funding Information later in the submission form are also	The authors have declared that no competing interests exist

declared in your Financial Disclosure statement.<br><br>View published research articles from *PLOS ONE* for specific examples.<br><br>**NO authors have competing interests**<br><br>Enter: *The authors have declared that no competing interests exist.*<br><br>**Authors with competing interests**<br><br>Enter competing interest details beginning with this statement:<br><br>*I have read the journal's policy and the authors of this manuscript have the following competing interests: [insert competing interests here]*<br><br>* typeset	
**Ethics Statement**<br><br>Enter an ethics statement for this submission. This statement is required if the study involved:<br><br>• Human participants<br>• Human specimens or tissue<br>• Vertebrate animals or cephalopods<br>• Vertebrate embryos or tissues<br>• Field research<br><br>Write "N/A" if the submission does not require an ethics statement.<br><br>General guidance is provided below. Consult the submission guidelines for detailed instructions. **Make sure that all information entered here is included in the Methods section of the manuscript.**	N/A

Powered by Editorial Manager® and ProduXion Manager® from Aries Systems Corporation

**Format for specific study types**<br><br>**Human Subject Research (involving human participants and/or tissue)**<br>• Give the name of the institutional review board or ethics committee that approved the study<br>• Include the approval number and/or a statement indicating approval of this research<br>• Indicate the form of consent obtained (written/oral) or the reason that consent was not obtained (e.g. the data were analyzed anonymously)<br><br>**Animal Research (involving vertebrate animals, embryos or tissues)**<br>• Provide the name of the Institutional Animal Care and Use Committee (IACUC) or other relevant ethics board that reviewed the study protocol, and indicate whether they approved this research or granted a formal waiver of ethical approval<br>• Include an approval number if one was obtained<br>• If the study involved *non-human primates*, add *additional details* about animal welfare and steps taken to ameliorate suffering<br>• If anesthesia, euthanasia, or any kind of animal sacrifice is part of the study, include briefly which substances and/or methods were applied<br><br>**Field Research**<br><br>Include the following details if this study involves the collection of plant, animal, or other materials from a natural setting:<br>• Field permit number<br>• Name of the institution or relevant body that granted permission	
**Data Availability**<br><br>Authors are required to make all data underlying the findings described fully available, without restriction, and from the time of publication. PLOS allows rare exceptions to address legal and ethical concerns. See the PLOS Data Policy and FAQ for detailed information.	Yes - all data are fully available without restriction

A Data Availability Statement describing where the data can be found is required at submission. Your answers to this question constitute the Data Availability Statement and **will be published in the article**, if accepted.<br><br>**Important:** Stating 'data available on request from the author' is not sufficient. If your data are only available upon request, select 'No' for the first question and explain your exceptional situation in the text box.<br><br>Do the authors confirm that all data underlying the findings described in their manuscript are fully available without restriction?	
**Describe where the data may be found in full sentences. If you are copying our sample text, replace any instances of XXX with the appropriate details.**<br><br>• If the data are **held or will be held in a public repository**, include URLs, accession numbers or DOIs. If this information will only be available after acceptance, indicate this by ticking the box below. For example: *All XXX files are available from the XXX database (accession number(s) XXX, XXX.).*<br>• If the data are all contained **within the manuscript and/or Supporting Information files**, enter the following: *All relevant data are within the manuscript and its Supporting Information files.*<br>• If neither of these applies but you are able to provide **details of access elsewhere**, with or without limitations, please do so. For example:<br><br>*Data cannot be shared publicly because of [XXX]. Data are available from the XXX Institutional Data Access / Ethics Committee (contact via XXX) for researchers who meet the criteria for access to confidential data.*<br><br>*The data underlying the results presented in the study are available from (include the name of the third party*	All relevant data are within the manuscript and its Supporting Information files.

and contact information or URL).<br>• This text is appropriate if the data are owned by a third party and authors do not have permission to share the data.<br><br>* typeset	
Additional data availability information:	

Powered by Editorial Manager® and ProduXion Manager® from Aries Systems Corporation

Manuscript Click here to access/download;Manuscript;Manuscript.docx

Title

Chemotaxis response and age-stage, two-sex life table of the *Cheilomenes sexmaculata* (Fabricius) (coccinellidae: coleoptera) against different aphid species

Authors

Hafiz Muhammad Safeer[1], Aimen Ishfaq[1], Syed Muhammad Zaka[1*] Alia Tajdar[2], Ahmad Saood[1], Zuraiz Ali Shah[1], Muhammad Shah Zaib[1], Khalid Abbas[1], Adeel Mukhtar [1], Muhammad Usama Altaf[1]

Affiliations

[1] Department of Entomology, Faculty of Agricultural Sciences and Technology, Bahauddin Zakariya University, Multan, Pakistan.

[2] College of plant protection, China Agricultural University, Beijing, China.

Corresponding author address

* Dr. Syed Muhammad Zaka

Email ID: zaka_ento@bzu.edu.pk

Contact Number: 00923127376838

ABSTRACT

The *Cheilomenes sexmaculata* (Fabricius) (Coleoptera: Coccinellidae), is one of the most beneficial and identifiable predators of numerous soft-bodied and sucking insect pests of several crops. Biological parameters and olfactory response of *C. sexmaculata* were investigated under laboratory conditions by providing three different aphid species i.e., mustard aphid (*Lipaphis erysimi*), citrus black aphid (*Toxoptera citricida*), and peach aphid (*Diuraphis noxia*) as a food source. The developmental period of immature stages of *C. sexmaculata* was shorter on *D. noxia* as compared to other aphid species. The adult longevities were longer on *D. noxia* and *T. citricida* while shorter on *L. erysimi*. Female fecundity was highest on *D. noxia* while lowest on *L. erysimi*. Life table parameters i.e., intrinsic rate of increase (*r*), finite rate of increase (λ), net reproductive rate (*R*o), and gross reproductive rate (*GRR*) were maximum on *D. noxia* while minimum on *L. erysimi*. The mean generation time *C. sexmaculata* was 20.90, 23.69, and 26.2 days on *D. noxia*, *L. erysimi*, *T.* and *citricida,*, respectively. These findings were further confirmed from the olfactory experiment where *D. noxia* proved to be the most preferred prey. This study provides necessary information for mass-rearing of *C. sexmaculata*.

Key words.

Zigzag beetle; *Diuraphis noxia; Toxoptera citricida; Lipaphis erysimi*; life table; olfaction

Introduction

Aphid (Hemiptera: Aphididae) is a polyphagous pest of many agricultural and horticultural crops (1). It is widely distributed in East Asia, North America (2), Europe (3), and Pakistan (4). Both nymphs and adults (5) of the aphid have great economic importance as they cause direct

damage to all plant parts by sucking cell sap (6), secret honeydew, (7) on which sooty mold develops and cause indirect damage by injecting toxic saliva along with different viruses (Pea enation mosaic virus and Bean leafroll virus) to plants (8, 9). Persistent feeding of aphid population to host plant cause the different losses like stunted plant growth, drying of leaves, decrease in photosynthesis that results in the reduction of yield (10). Aphids can complete more than 10 generations in a year on different hosts (11)

Coccinellids have a wide range of hosts and lived in a variety of environments. Predaceous coccinellids have great economic importance as the grubs and adults feed on many phytophagous soft-bodied arthropods, such as whiteflies, aphids, thrips, jassids, mealy bugs, psyllids, leafhoppers, scale insects, and mites, which are the destructive pests of agro- and forest ecosystems (12). The predatory beetles *C. sexmaculata* are found in all Asian countries. The zigzag beetle *C. sexmaculata* is the most common species in Pakistan (13), India, (14), China (15), Indonesia (16), and Japan (17).

There are different control methods (cultural, mechanical, biological, and chemical) used to suppress the pest population in the field. Chemical control is one of them, in which different types of insecticides like flonicamid, sulfoxaflor, and afidopyropen are used for the management of aphids (18). Insecticidal control is not only expensive but also harmful to the natural environment and causes several health problems (19). Continuous applications of pesticides develop phytotoxicity in plants and destroy natural enemies such as predators, parasitoids, microorganisms, and pollinators (20, 21). The use of biological control agents for the suppression of the aphid population is the best alternative control tactic to highly toxic insecticides (22). The Coccinellids are successful predators over other predators having resistance to several insecticides (23).

The influence of different aphid species affects the biology and predatory efficiency of the coccinellid beetle. The different aphid species have a significant effect on the development of *C. sexmaculata* (24). Similarly, different aphid species had a significant influence on the predation as well as life table attributes of *C. sexmaculata* (25). Likewise, on other coccinellid beetles, similar significant responses of predatory coccinellids against different prey species have been reported (26). There was a lot of previous research published to evaluate the effect of different aphid species on life tables and the biology of coccinellids (27, 28). Further, olfactory studies were used extensively to evaluate the chemotaxis responses of the ladybird beetle towards its prey (29-31) but there is no report on this aspect of *C. sexmaculata*. Keeping in view the importance of aphids and ladybird beetles, current work had been designed to evaluate the developmental and behavioral responses of immatures and adults of *C. sexmaculata* by providing different aphids species, which has not been previously reported.

Material and methods

Collection and mass-rearing of predator

The field population of *C. sexmaculata* was collected by using an aspirator and camel hairbrush from *Calotropis procera* present around the Agricultural fields, Bahauddin Zakariya University, Multan, Pakistan. Field collected adults and larvae were placed separately in rearing plastic jars (8×8×14 cm) reared till homogenous population was achieved under control conditions (25±1°C and 70±2% R.H. and 16L:8D h) (32). Aphids were used as a food source for both larvae and adults. The jars were closed with the muslin cloth for ventilation and to avoid contamination. Eggs were collected from adults rearing jars and placed in a petri dish (6cm diameter), having moist filter paper at the base (33).

Collection and rearing of prey

The collection of three different aphid species Mustard aphid (*Lipaphis erysimi*), Citrus black aphid (*Toxoptera ~~C~~itricida*), and Peach aphid (*Diuraphis noxia*) was done from their respective hosts i.e., *Brassica campestris, Citrus clementina,* and *Triticum aestivum*. These aphid species were reared on their respective host plants separately in cages (43.75×43.75×50 cm) under laboratory-controlled conditions and fresh diets were provided on daily basis (12).

Biological parameters

Eggs of *C. sexmaculata* were taken from a homogeneous population and placed singly in the petri dish (6cm diameter). After hatching, three aphid species were provided throughout their whole life and each aphid species was considered as a treatment. There were thirty replications in each treatment. The counted number of aphids were released in each replication and the number of aphids consumed was recorded daily till the formation of the pupa. Different larval instars of *C. sexmaculata* were identified by the presence of exuvium. After the emergence of adults, five pairs (each pair represent one replication) of each treatment were placed in plastic jars (8×8×14 cm) separately. Pre-oviposition, oviposition, post-oviposition, adult survival rate, male and female longevity were recorded (34).

Olfactometer Experiment

The olfactory response of all four instars and adults (male and female) of *C. sexmaculata* against two different aphid species (*D. noxia* and *L. erysimi*) was determined by using a four-arm olfactometer as described by Vet et al (35). The olfactory chamber was connected with four arms (each arm consist of three flasks having odor source, water and charcoal, respectively, followed

by an airflow meter) and a hole in the center for suction, the same hole was also used for entry of insect. The suction pump was connected at the central hole of the olfactory chamber and the flow of air was adjusted by air flow meter at 4 kpa/min. The olfactory chamber was divided into four regions (A, B, C and D), each region representing the specific odor received from a specific arm. The light source (12 KW LED bulb) was also hung at the center of the olfactory chamber (36). A black cloth was used around the whole olfactory apparatus to ensure the uniform light condition.

The larval instars (1st, 2nd, 3rd & 4th) and adults (male and female) of *C. sexmaculata* were placed singly in the olfactory chamber through central opening and monitored for 10 minutes (37). The retention time was recorded by using a stopwatch and the number of entries by visual counting. Data recording was started after 15 seconds (settlement time). The individuals who spent more than 50% time in the central region of the olfactory chamber were discarded. Three replications were performed of each instar as well as adults. The insect was considered as a choice insect when it remained 15 seconds or more in the order source region. Moreover, the direction of the apparatus was changed to 90° after every replication to remove the possible side effects on the insect behavior. The apparatus was washed with detergents and 70% ethanol after each replication and kept for drying to remove the contamination (29).

Statistical analysis

The biological parameters and olfactory response of larvae and adults of *C. sexmaculata* were analyzed by one-way analysis of variance (ANOVA) with the help of the statistical package SAS (38). The means of different treatments were compared by using the Least Significant Difference (LSD) at 5% probability.

TWO SEX-MS Chart was used to compute different population parameters of *C. sexmaculata* (39). The bootstrap technique of TWO SEX-MS Chart with 100,000 replications were performed to minimize the variations in the results (40). The recorded data were used to analyze the age-stage specific fecundity (f_{xj}, where x = age in days and j = stage), age-stage–specific survival rate (S_{xj}), age-stage reproductive value (V_{xj}), age-stage life expectancy(e_{xj}), age-specific fecundity (m_x), age-specific survival rate (l_x), age-specific net maternity ($l_x m_x$), and life table parameters like, λ, finite rate of increase r, intrinsic rate of increase R_0, net reproductive rate; and T, the mean generation (41).

In the age-stage, two-sex life table, the age-specific survival rate (l_x), age-specific fecundity (m_x), net reproductive rate (R_0), finite rate of increase (r), finite rate (λ), mean generation time (T) and life expectancy (e_{xj}) were calculated by following equations;

$$l_x=\sum_{j=1}^{k} S_{xj} \quad \text{………………………. ……..(1)}$$

$$m_x=\frac{\sum_{j=1}^{k} s_{xj} f_{xj}}{\sum_{j=1}^{k} s_{xj}} \quad \text{…………………………......(2)}$$

Where k is the number of stages.

$$R_0=\sum_{x=0}^{\infty} l_x m_x \quad \text{………………………….. (3)}$$

where the net reproductive rate is the mean number of offspring laid by the individual during its entire life span.

$$\sum_{x=0}^{\infty} e^{-r(x+1)} l_x m_x = 1 \text{ ....(4)}$$

Where Iterative bisection method was used to estimate and corrected the intrinsic rate of increase (r) with the Euler–Lotka equation (42).

$$\lambda = e^r \text{ .... (5)}$$

$$T = In^{R_0}/_r \text{ ....(6)}$$

Where mean generation time is defined as the length of time that a population needs to increase to R_0-fold of its population size at the stable age-stage distribution.

$$e_{xj} = \sum_{i=x}^{\infty} \sum_{y=j}^{\beta} s'_{iy} \text{ ....(7)}$$

Where life expectancy (e_{xj}) is the length of time that an individual of age x and stage j is expected to live (43).

Results

Biological parameters

The developmental period of each immature stage, adult longevity, and female fecundity of *C. sexmaculata* fed on three different aphid species (Table 1). The developmental period of the eggs was statistically the same for all tested aphid species. The developmental period of the first larval

instar was significantly shorter (F=19.35; $F_{2,87;}$ P<0.0001) when reared on *D. noxia* (0.93 days), followed by 1.60 days when fed on *T. citricida*, while the longest (2.50 days) duration was observed when fed on *L. erysimi*. The second instar larvae completed its development significantly (F = 14.21; $F_{2,87}$; P<0.0001) shorter (00 days) on *D. noxia*, while reared on *T. citricida* completed its development in 1.73 days and the longest development time (2.67 days) was observed on *L. erysimi*. A shorter developmental period (1.40 days) was observed in third instar larvae when *D. noxia* was provided as food, followed by 1.90 days on *T. citricida* and longer development time i.e., 2.47days on *L. erysimi* (F=3.24; $F_{2,87;\ P}$ = 0.0463). Fourth instars and pupae of the *C. sexmaculata* shared the same statistical rank on all aphid species. All three aphid species have significant (F = 6.00; $F_{2,87;}$ P=0.0043) influenced on the total duration of immature stages. The total developmental duration of immature stages was longer on *L. erysimi* (13.07 days) but shorter on *D. noxia* (8.20 days).

A significant difference in adult longevity of males and females was observed when different aphid species were provided to them as food. Males and females lived significantly shorter (F = 112; $F_{2,24,}$ P=0.0001 and F = 158.18; $F_{2,1,}$ P=0.0001, respectively) when fed on *L. erysimi* (11.11 and 12.13 days, respectively) and longer on *D. noxia* (26days) and *T. citricida* (32days), respectively. When the beetles fed on different aphid's species showed the significant effect on pre-oviposition period (F = 9.00; $F_{2,35,}$ P=0.0031) and post oviposition period (F = 93.31; $F_{2,87}$; P=0.0001). The oviposition period was maximum (10.25 and 9.12 days) when *C. sexmaculata* fed on *D. noxia* and *T. citricida*, respectively, while the minimum was recorded on *L. erysimi* (5.00 days) (F = 29.42; $F_{2,35;}$ P<0.0001).

Fecundity of *C. sexmaculata* varied significantly (F = 18.55; $F_{2,35;}$ P<0.0001) on different aphid species. Maximum fecundity was recorded when *D. noxia* (265.91eggs/female) and *T.*

citricida (216.60eggs/female) were given as food while minimum fecundity was recorded on *L. erysimi* (64.88 eggs/female) (Table 1).

Table 1. Development period (mean ± SE) of *M. sexamaculatus* fed on three aphid species

**Developmental Stages/ Ovipositioning**	***Lipaphis erysimi***	***Toxoptera citricida***	***Diuraphis noxia***	**Statistical parameters**		
				**F-value**	**df**, Edf*****	**P-value**
Eggs(days)	1.80a ± 0.11	1.73a ± 0.13	1.73a ± 0.13	0.1	2,87	0.9077
First instar(days)	2.50a ± 0.24	1.60b ± 0.16	0.93c ± 0.08	19.35	2,87	<0.0001
Second instar(days)	2.67a ± 0.31	1.73b ± 0.19	1.00c ± 0.11	14.21	2,87	<0.0001
Third instar(days)	2.47a ±0.37	1.90ab ± 0.22	1.40b ± 0.20	3.24	2,87	0.0463
Fourth instar(days)	2.00± 0.33	1.70 ± 0.26	1.60 ± 0.21	0.64	2,87	0.5317
Pupa(days)	1.63 ± 0.29	1.93 ± 0.30	1.53 ± 0.22	0.62	2,87	0.5409
Total period from egg to pupae(days)	13.07a ± 1.22	10.53ab ± 1.00	8.20b ± 0.68	6.00	2,87	0.0043
Male longevity(days)	11.11b ±1.20	26.22a ± 0.94	26.00a ± 0.85	112	2,24	<0.0001
Female longevity(days)	12.13b ± 1.15	32.0a ± 0.66	32.25a ± 0.70	158.18	2,21	<0.0001
Pre-oviposition period(days)	4.00b ± 0.42	6.25a ± 0.59	5.13ab ± 0.71	9.00	2,35	0.0031
Oviposition period(days)	5.00b ± 0.53	9.12a ± 0.29	10.25a ± 0.72	29.42	2,35	<0.0001
Post-oviposition period(days)	3.13b ± 0.72	16.50a ± 0.88	16.63a ± 0.54	93.31	2,35	<0.0001
Fecundity (eggs/female)	64.88b ± 9.97	216.6a ± 15.05	265.91a ± 33.41	18.55	2,35	<0.0001

*Mean followed by different letters in the same row are significantly different ($P < 0.05$)

**df stands for Degree of freedom

***Edf stands for Error degree of freedom

Life table parameters i.e., *r*, λ, and *R*o (0.21 d^{-1},1.24 d^{-1} and 97.5 offspring, respectively) were higher on *D. noxia* as compared to other tested aphid species. Mean generation time (*T*) was higher (26.20 $^{d-1}$) on *T. citricida* than on *L. erysimi* (23.69 d^{-1}) while the lowest was observed on *D. noxia* (20.90 d^{-1}). The maximum gross reproductive rate (*GRR*) of the *C. sexmaculata* was 146.2 offspring when fed on *D. noxia* followed by 114 and 63.08 offspring on *T. citricida* and *L. erysimi* respectively (Table 2).

Table 2. Life table parameters of *M. sexamaculatus* reared on three tested aphid species

Parameters	Values of life table parameters on aphid species		
	Lipaphis erysimi	***Toxoptera citricida***	***Diuraphis noxia***
r (d^{-1})	0.12	0.16	0.21
λ (d^{-1})	1.13	1.17	1.24
Ro (Offspring individual ^{1})	17.3	72.2	97.5
T (d)	23.69	26.2	20.90
GRR(Offspring)	63.08	114	146.2

**r* = intrinsic rate of increase, *λ* = finite rate of increase, *Ro* = net reproductive rate *T* = mean generation time, *GRR* = the gross reproductive rate.

The eggs and second instar exhibited the survival probability (S_{xj}) 1 and 0.8667, respectively against all provided aphid species. Similar survival probability of first and third instars of *C. sexmaculata* was observed i.e., 0.8667 and 0.8333, respectively, on *D. noxia* and *T. citricida* while on *L. erysimi* survival probability was 0.93 and 0.8, respectively for both first and third instars. In the case of the fourth instar survival probability (0.8333) was observed on *D. noxia* followed by 0.8 on *T. citricida* while 0.7 was observed on *L. erysimi.* The pupae of *C. sexmaculata* exhibited the survival probability (0.7333) on *D. noxia* followed by 0.6333 and 0.6 on *T. citricida* and *L. erysimi*, respectively. A similar trend of survival probability was observed in females i.e., 0.3667, 0.3333, and 0.2667 in connection with *D. noxia*, *T. citricida,* and *L. erysimi*, respectively. Survival probabilities (0.3) of males were the same with all tested aphid species (Fig. 1).

Fig 1. Age-stage–specific survival rate (S_{xj}) of *C. sexmaculata* fed on three aphid species

Cheilomenes sexmaculata showed the highest survival rate (*lx*) on *D. noxia* and lowest on *L. erysimi*. The age-stage-specific female fecundity (*fx7*) of *C. sexmaculata* was maximum on *T. citricida* (34.1 eggs at the age 27 days), but minimum on *L. erysimi* (16 eggs at the age 28 days). Similarly, age-specific fecundity (*m*x) and Age-specific net maternity (*lxmx*) highest on *T. citricida*, but lowest on *L. erysimi* (Fig.2).

Fig 2. Age-specific survival rate (*lx*), age-stage–specific fecundity (*fxj*), age-specific fecundity (*mx*), and age-specific maternity (*lxmx*) of *C. sexmaculata* fed on three aphid species

The value of age-stage–specific reproductive rates (V_{xj}) was highest in the case of *T. citricida* (116.44 eggs at the age 23 days) and lowest on *L. erysimi* (48.02 eggs at the age 28 days) (Fig.3).

Fig 3. Age-stage–specific reproductive rate (V_{xj}) of *C. sexmaculata* fed on three aphid species

Maximum life expectancy (e_{xj}) 33.78 was observed at the pupal stage after 10 days in the case of *T. citricida*. In the case of *D. noxia,* a maximum life expectancy 34.82 was observed in the female stage at age 7 days. Maximum life expectancy 20.76 was observed in the third larval instar at age 5 days in the case of *L. erysimi* (Fig.4).

Fig 4. Age-stage–specific life expectancy (*exj*) of *C. sexmaculata* fed on three aphid species

Olfaction

Olfactory response of larval instars of C. sexmaculata against D. noxia and L. erysimi

The first instar of *C. sexmaculata* spent significantly (p<0.0001) maximum time (205.0 sec) in the region having the odor of *D. noxia* as compared to blank. While, in the case of *L. erysimi*, a non-significant (p=0.0620) response was observed to all four regions of the olfactometer. Significantly (p< 0.0064) the highest time (202.3 sec) was spent by the second instar in the region having volatiles of *D. noxia* than control. Similarly, a significant (p=0.0075) response was observed against *L. erysimi*. The third instar of *C. sexmaculata* spent significantly (p=0.0215) more time (188.0 sec) in the region where volatiles of *D. noxia* was provided than control, while in the case of *L. erysimi*, non-significant (p=0.1405) attraction was observed. The fourth instar of *C. sexmaculata* spent significantly (p=0.0039) maximum time (206.3 sec) in the region having the

odor of *D. noxia* as compared to blank. Moreover, a non-significant (p=0.0878) response was observed in the case of *L.erysimi* (Fig. 5).

Fig 5. Mean time spent (sec) and the number of entries in each region of a four-arm olfactometer by different instars of *C. sexmaculata* exposed to *D. noxia* (a, b, c & d) and *L. erysimi* (e, f, g & h)

Olfactory response of male and female of C. sexmaculata against D. noxia and L. erysimi

The male of *C. sexmaculata* spent significantly (p=0.004) maximum time (213.3 sec) in the region having the odor of *D. noxia* as compared to blank while, in the case of *L. erysimi,* non-significant (p= 0.1094) response was observed. The significantly (p<0.0001) maximum time (239.0 sec) spent by the female of *C. sexmaculata* in the region having the odor of *D. noxia* as compared to blank while, in the case of *L. erysimi,* non-significant (p=0.0753) response was observed (Fig 6).

Fig 6. Mean time spent (sec) and the number of entries in each region of a four-arm olfactometer by different instars of *C. sexmaculata* exposed to *D. noxia* (a & b) and *L. erysimi* (c & d)

Discussion

The purpose of this study was to examine how different aphid species affect the development, survival rate, and fecundity of *C. sexmaculata*. The outcomes showed that the availability and quality of prey influenced the development of *C. sexmaculata*. These findings are in consistent with the biological study of *C. sexmaculata* on different aphid species in which the nature and quality of the prey change the feeding behavior of predators and alter their development, survival rate, and fecundity (13). The findings of current research evidenced that the development

period was delayed in the case of *L. erysimi* as compared to other aphid species, which showed that prey quality effects predator development. A similar finding was observed in a study where Predator development is delayed by the deficient quality and inadequate amount of prey, while predator development is encouraged by the nutritious quality and adequate amount of prey (44). The developmental period of immatures of coccinellid fluctuated greatly by feeding on different aphid species, this is comparable with the findings showed that the longest preadult developmental period of *C. septempunctata* was observed on *A. craccivora* while the shortest was seen on *M. persicae* (27). The results of the present study showed that adults' longevities were longer on *D. noxia* while shorter on *L. erysimi*. It correlates to a biological study of *C. sexmaculata* on different seven aphid species. The longer adult longevity of predatory beetle was observed on *A. craccivora* while shorter on *L. erysimi* than other tested aphid species (26). A shorter developmental period of *C. sexmaculata* was observed on *L. erysimi* in the present study. These findings are conflicted with the study that was performed on *C. septempunctata* and proved that the longer developmental period was observed on *L. erysimi* while shorter on *M. rosae* (45). The variations occurred in the results of different studies due to the different biotic (predator, prey and plant species) and abiotic (mass-rearing conditions of predator and prey) factors.

The quantity and quality of aphid species have a direct effect on female fecundity. The highest fecundity was observed when *D. noxia* was provided as food and the lowest fecundity was observed on *L. erysimi*. Previous studies also proved that the dietary quality of prey influenced predator fecundity (46). In the current study, the highest female fecundity was observed on *D. noxia* while the lowest was on *L. erysimi*. The findings of the current study are correlated with the previous study indicated that the maximum lifetime fecundity of *Anegleis cardoni* was found on *A. gossypii* and the minimum on *L. erysimi* (47).

There were many difficulties associated with the traditional life tables that explained the female population but neglected the male populations and stage variation among individuals and both sexes. A modern life table (age-stage two-sex life table) was applied in the present study to overcome the difficulties (the variations among age-specific fecundity and age-specific survival rate) connected to traditional life tables. Life table parameters (l_x and m_x) were used for the consideration of the survival rate of male and stage variation among individuals. A detailed description of the issues and errors with female age-specific life tables was provided (43, 48).

Life table parameters are greatly affected by various factors i.e., the quality and nature of host plant and prey species, and controlled conditions of the laboratory (49, 50). The outcomes of the present study exhibited that maximum R_0, r, λ, and *GRR* parameters were recorded on *D. noxia* and minimum on *L. erysimi,* while *T* was maximum on *T. citricida* followed by *L. erysimi* and minimum on *D. noxia*. These observations are similar to the study that outcomes revealed that R_0, r, λ, and *GRR* were highest on *A. gossypii* and lowest on *L. erysimi* while *T* was maximum on *L. erysimi* and minimum on *A. gossypii* (13, 47).

The survival rate of *C. sexmaculata* was highest when fed on *D. noxia*, according to the age-stage-specific survival rate (S_{xj}) curves. The findings are comparable to the study of *C. sexmaculata* in which the highest survival rate was also recorded on *D. noxia* than other aphid species (13). The highest age-stage-specific female fecundity and Age-specific survival rate of *C. sexmaculata* were observed on *D. noxia* while the lowest age-stage-specific female fecundity and Age-specific survival rate were recorded on *L. erysimi*. The results of the previous study correlated with current research that *L. erysimi* exhibited the lowest age-stage-specific female fecundity with *H. convergens,* while the age-specific survival rate of *H. convergens* was maximum on *L. erysimi,* which is contrary to the present result (28). The difference in the results depends on the species of

predator and prey. The age-stage–specific reproductive rate (v_{xj}) provides precise information of an individual's contribution to population increase in the future at age *x* and stage *j*. The age-stage–specific reproductive rate (v_{xj}) was highest in the case of *T. citricida* and lowest on *L. erysimi.* Similar results were found in the study of *H. convergens* on different aphid species (28). The results showed that the life expectancy of individuals decreased with the increase of age. This is consistent with a study on *C. flavocapitis* under laboratory conditions, which found that life expectancy declined with age (51) The age-stage two-sex life table described the biological parameters of all stages of *C. sexmaculata.* This study concluded that prey species significantly affected the biological parameters and *D.noxia* is consider as a best prey for the mass rearing of predatory beetle (*C.* sexmaculata) under laboratory conditions. Moreover, future studies should emphasize on the field efficacy and application of the *M. sexmaculatus* for the control of different aphids.

Funding

No funding was available for this research work.

Conflict of interest

The authors declare that they have no known competing financial interests or personal relationships that could have appeared to influence the work reported in this paper.

Acknowledgements

The authors wish to acknowledge the Department of Entomology, for coordinating in the present research work and undergraduate students for helping in experiments. Further, authors are thankful to undergraduate students for helping during research work.

Authors contribution

HMS: Conceptualization, Investigation, writing - review and editing. AI: Investigation, Formal analysis, editing. SMZ: Project administration, Supervision, editing. AJ: analysis, editing, review. AS: Investigation. ZAS: Insect collection, Investigation. MSZ: Data analysis, editing. KA: Data analysis, editing. AM: Data collection, Rearing, Insect collection. MUA: Rearing, Field Collection .

References

1. Guidolin AS, Cônsoli FL. Influence of host plant on oligophagous and polyphagous aphids, and on their obligate symbiont titers. Biologia. 2020; 75: 71-81.
2. Kim H, Hoelmer KA, Lee S. Population genetics of the soybean aphid in North America and East Asia: test for introduction between native and introduced populations. Biol Invasions. 2017; 19: 597-614.
3. Wieczorek K, Fulcher TK, Chłond D. The composition of the aphid fauna (Insecta, Hemiptera) of the Royal Botanic Gardens, Kew. Sci Rep. 2019; 9: 1-14.
4. Maryam S, Sandhu AA, Bodlah I, Aziz MA, Aihetasham A. Contribution to aphid's fauna of Gujranwala (Punjab), Pakistan. Punjab Univ J Zool. 2019; 34: 09-16.
5. Coppola M, Manco E, Vitiello A, Di Lelio I, Giorgini M, Rao R, et al. Plant response to feeding aphids promotes aphid dispersal. Entomol Exp Appl. 2018; 166: 386-94.
6. Sathe TV, Gophane A, Shendage N. Colour attractivity and occurrence of some cell sap sucking pests on crop plants. Biolife. 2015; 3: 540-6.
7. Fischer MK, Völkl W, Hoffmann KH. Honeydew production and honeydew sugar composition of polyphagous black bean aphid, *Aphis fabae* (Hemiptera: Aphididae) on various host plants and implications for ant-attendance. Eur J Entomol. 2005; 102: 155-60.

8. Donnelly R, Cunniffe NJ, Carr JP, Gilligan CA. Pathogenic modification of plants enhances long- distance dispersal of nonpersistently transmitted viruses to new hosts. Ecology. 2019; 100: e02725.

9. Paudel S, Bechinski EJ, Stokes BS, Pappu HR, Eigenbrode SD. Deriving economic models for pea aphid (Hemiptera: Aphididae) as a direct-pest and a virus-vector on commercial lentils. J Econ Entomol. 2018; 111: 2225-32.

10. Blackman RL, Eastop VF. Aphids on the world's crops: an identification and information guide. John Wiley & Sons Ltd; 2000.

11. Iversen T, Harding S. Life table parameters affecting the population development of the woolly beech aphid, *Phyllaphis fagi*. Entomol Exp Appl. 2007; 123: 109-17.

12. Khoso AG, Khan M, Ahmed S. Feeding Preference of Larvae and Adults of Zigzag Beetle on Sucking Insect Pests of Brinjal Under Lab. Condition at Tando Jam. Int J Appl Sci Biotechnol. 2019; 7: 27-30.

13. Abbas K, Zaib MS, Zakria M, Hani U-e, Zaka SM, Ane MN-u. *Cheilomenes sexmaculata* (Coccinellidae: Coleoptera) as a potential biocontrol agent for aphids based on age-stage, two-sex life table. Plos one. 2020; 15: e0228367.

14. Kumar S, Ahmad ME, Rakhshan R. Influence of Prey Species on Feeding Preference, Post-Embryonic Development and Life Span of *Cheilomenes Sexmaculata* (Fabricius). European Scientific Journal. 2016; 12: 403-10.

15. Huang J, Xu Y-J, Lu Y-Y, Liang G-W, Zeng L. Effects of the invasive ant *Solenopsis invicta* (Hymenoptera: Formicidae) on *Menochilus sexmaculatus* (Coleoptera: Coccinellidae) as a predators of *Aphis craccivora* (Hemiptera: Aphididae) in laboratory conditions. Sociobiology. 2011; 57: 565-74.

16. Setiawati W, Gunaeni N, Uhan T, Hasyim A. Potency of predator (*Menochilus sexmaculatus*) augmentation for white fly (*Bemisia tabaci*) management and its effect on gemini virus infestation on tomato. Indones J Agric Sci. 2012; 13: 18-26.

17. Kawakami Y, Yamazaki K. Disappearance of summer collection records for *Menochilus sexmaculatus* (Coleoptera: Coccinellidae) during climate warming. J Nat Hist. 2017; 51: 1015-20.

18. Slusher EK, Cottrell T, Acebes-Doria AL. Effects of Aphicides on Pecan Aphids and Their Parasitoids in Pecan Orchards. Insects. 2021; 12: 1-12.

19. Pimentel D. Environmental and economic costs of the application of pesticides primarily in the United States In Integrated Pest Management: Innovation-Development Process 89–111. Springer; 2009.

20. Bommarco R, Miranda F, Bylund H, Björkman C. Insecticides suppress natural enemies and increase pest damage in cabbage. J Econ Entomol. 2011; 104: 782-91.

21. Shahid M, Khan MS, Ahmed B, Syed A, Bahkali AH. Physiological disruption, structural deformation and low grain yield induced by neonicotinoid insecticides in chickpea: A long term phytotoxicity investigation. Chemosphere. 2021; 262: 128388.

22. Shonga E, Getu E. Efficacy of plant derived and synthetic insecticides against cabbage aphid, *Brevicoryne brassicae* (L.)(Homoptera: Aphididae) and their effect on coccinellid predators. Ethiop J Sci. 2021; 44: 27-37.

23. Wu G, Wang Y, Wang JN, Chen XZ, Hu QX, Yang YF, et al. Vitality and stability of insecticide resistance in adult *Propylaea japonica* (Coleoptera: Coccinellidae). J Insect Sci. 2018; 18: 1-16.

24. Pandi G, Paul B, Vivek S, Shankarganesh K. Feeding potential and biology of Coccinellid predator *Cheilomenes sexmaculata* (Fabricius)(Coleoptera) on aphid hosts. Indian J Entomol. 2012; 74: 388-93.

25. Solangi BK, Hullio MH, Baloch N. Biological parameters and prey consumption by zigzag beetle *Menochilus sexmaculatus* Fab. against *Rhopalosiphum maidis* Fitch, *Aphis gossypii* Glov. and *Therioaphis trifolii* Monell. Sarhad J Agric. 2007; 23: 1097-101.

26. Omkar, Bind R. Prey quality dependent growth, development and reproduction of a biocontrol agent, *Cheilomenes sexmaculata* (Fabricius)(Coleoptera: Coccinellidae). Biocontrol Sci Technol. 2004; 14: 665-73.

27. Farooq M, Shakeel M, Iftikhar A, Shahid MR, Zhu X. Age-stage, two-sex life tables of the lady beetle (Coleoptera: Coccinellidae) feeding on different aphid species. J Econ Entomol. 2018; 111: 575-85.

28. Arshad M, Ullah MI, Shahid U, Tahir M, Khan MI, Rizwan M, et al. Life table and demographic parameters of the coccinellid predatory species, *Hippodamia convergen*s Guérin-Méneville (Coleoptera: Coccinellidae) when fed on two aphid species. Egypt J Biol Pest Control. 2020; 30: 1-8.

29. Raymond B, Darby A, Douglas A. The olfactory responses of coccinellids to aphids on plants. Entomol Exp Appl. 2000; 95: 113-7.

30. Francis F, Lognay G, Haubruge E. Olfactory responses to aphid and host plant volatile releases:(E)-β-farnesene an effective kairomone for the predator *Adalia bipunctata*. J Chem Ecol. 2004; 30: 741-55.

31. Alhmedi A, Haubruge E, Francis F. Identification of limonene as a potential kairomone of the harlequin ladybird *Harmonia axyridis* (Coleoptera: Coccinellidae). Eur J Entomol. 2010; 107: 541-8.

32. Zhao J, Li S, Gao X-W, Zhang F, Wang S. Comparison of life tables of *Cheilomenes sexmaculata* (Coleoptera: Coccinellidae) under laboratory and greenhouse conditions. J Econ Entomol. 2015; 108: 1700-7.

33. Khan A. Functional response of *Adalia tetraspilota* (Hope)(Coleoptera: Coccinellidae) on cabbage aphid, *Brevicoryne brassicae* (L.)(Hemiptera: Aphididae). J Biol Control. 2009; 23: 243–8.

34. Farooq M, Zhu X, Shakeel M, Iftikhar A, Shahid MR, Saeed N, et al. Comparative analysis of the demographic parameters of seven spotted ladybird beetle (Coleoptera: Coccinellidae) reared on various host aphid species. PeerJ. 2020; 8: e8313.

35. Vet LE, Lenteren Jv, Heymans M, Meelis E. An airflow olfactometer for measuring olfactory responses of hymenopterous parasitoids and other small insects. Physiol Entomol. 1983; 8: 97-106.

36. Rashedi A, Rajabpour A, Rasekh A, Zandi-Sohani N. Interactions between host plant, Aphis fabae, and its natural enemies, Orius albidipennis and Lysiphlebus fabarum in a tritrophic system. J Asia-Pacif Entomol. 2019; 22: 847-52.

37. Hilker M, Kobs C, Varama M, Schrank KJJoEB. Insect egg deposition induces Pinus sylvestris to attract egg parasitoids. J Exp Biol. 2002; 205: 455-61.

38. SAS institute Inc. Institute s. SAS 9.1 for Windows. SAS Institute Cary, NC; 2002. 2002:

39. Chi H. TIMING-MSChart: a computer program for the population projection based on age-stage, two-sex life table. 2016.

40. Tibshirani RJ, Efron B. An introduction to the bootstrap. Monog Stat Appl Probab. 1993; 57: 1-436.

41. Huang YB, Chi H. Age stage, two sex life tables of *Bactrocera cucurbitae* (Coquillett)(Diptera: Tephritidae) with a discussion on the problem of applying female age-specific life tables to insect populations. Insect Sci. 2012; 19: 263-73.

42. Goodman D. Optimal life histories, optimal notation, and the value of reproductive value. The American Naturalist. 1982; 119: 803-23.

43. Chi H, Su H-Y. Age-stage, two-sex life tables of *Aphidius gifuensis* (Ashmead)(Hymenoptera: Braconidae) and its host *Myzus persicae* (Sulzer)(Homoptera: Aphididae) with mathematical proof of the relationship between female fecundity and the net reproductive rate. Environ Entomol. 2006; 35: 10-21.

44. Snyder WE, Joseph SB, Preziosi R, Moore AJ. Nutritional benefits of cannibalism for the lady beetle *Harmonia axyridis* (Coleoptera: Coccinellidae) when prey quality is poor. Environ Entomol. 2000; 29: 1173-9.

45. Ali A, Rizvi PQ. Development and predatory performance of *Coccinella septempunctata* L.(Coleoptera: Coccinellidae) on different aphid species. J Biol Sci. 2007; 7: 1478-83.

46. Sarwar M. Influence of host plant species on the development, fecundity and population density of pest *Tetranychus urticae Koch* (Acari: Tetranychidae) and predator *Neoseiulus pseudolongispinosu* s (Xin, Liang and Ke)(Acari: Phytoseiidae). N Z J Crop Hortic Sci. 2014; 42: 10-20.

47. Omkar KG, Sahu J. Performance of a predatory ladybird beetle, *Anegleis cardoni* (Coleoptera: Coccinellidae) on three aphid species. Eur J Entomol. 2009; 106: 565-72.

48. Huang YB, Chi H. Life tables of *Bactrocera cucurbitae* (D iptera: T ephritidae): with an invalidation of the jackknife technique. J Appl Entomol. 2013; 137: 327-39.

49. Rana L, Mainali R, Regmi H, Rajbhandari B. Effect of Different Preys on Certain Biological Characteristics of Green Lacewing, *Chrysoperia carnea* (Stephens)(Neuroptera: Chrysopidae) under Laboratory Conditions. J Plant Protect Soci. 2020; 6: 108-17.

50. Lei X, Li D, Li Z, Zalom FG, Gao L, Shen Z. Effect of host plants on developmental time and life table parameters of *Carposina sasakii* (Lepidoptera: Carposinidae) under laboratory conditions. Environ Entomol. 2012; 41: 349-54.

51. Bailey R, Chang N-T, Lai P-Y. Two-sex life table and predation rate of *Cybocephalus flavocapitis* Smith (Coleoptera: Cybocephalidae) reared on *Aulacaspis yasumatsui* Takagi (Hemiptera: Diaspididae), in Taiwan. J Asia-Pacif Entomol. 2011; 14: 433-9.

Figure 1

Click here to access/download;Figure;Survival stage Fig 1.tiff

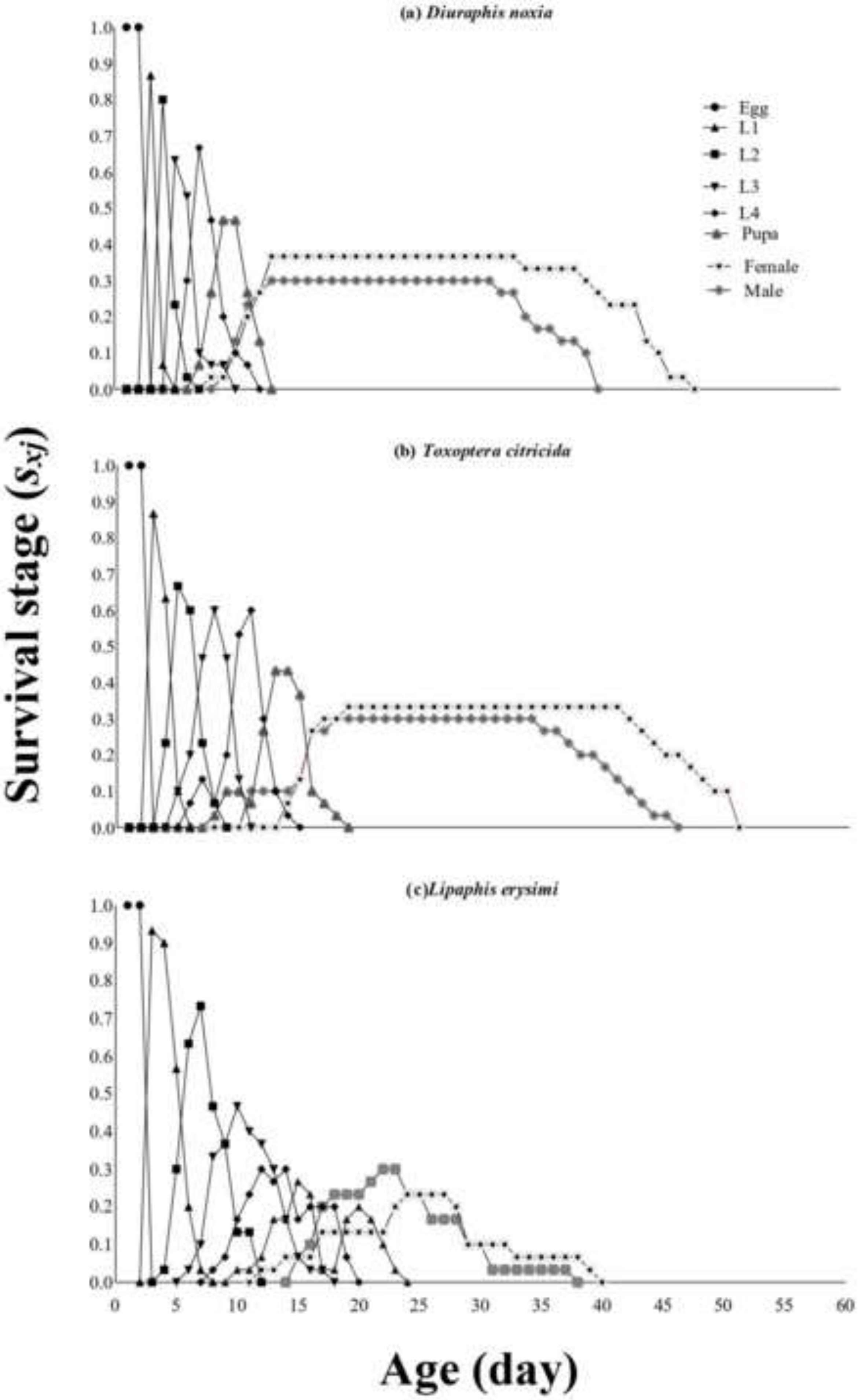

Figure 2

Click here to access/download;Figure;Survival rate Fig 2.tiff

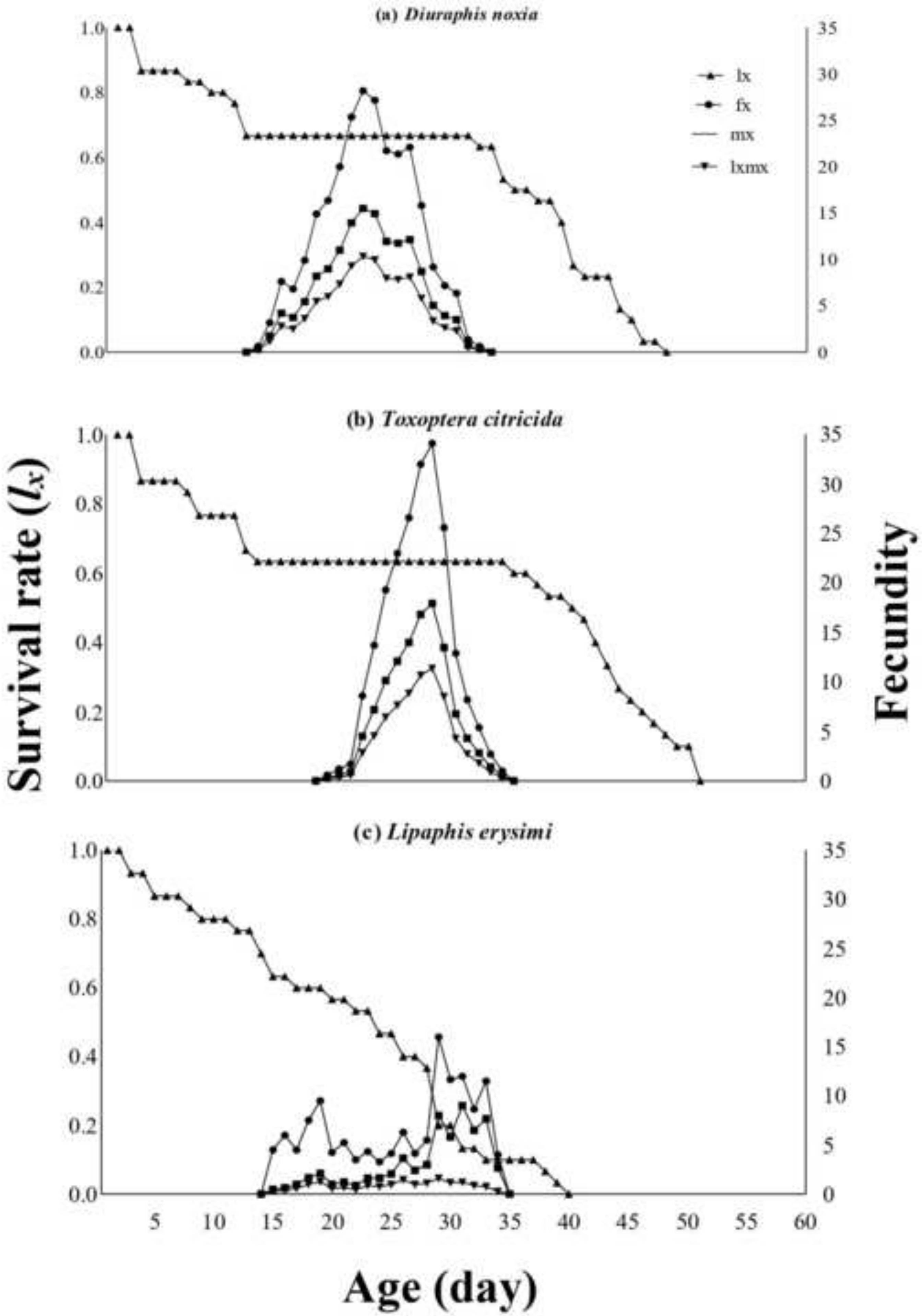

 Click here to access/download;Figure;Repridductive value Fg 3.tiff

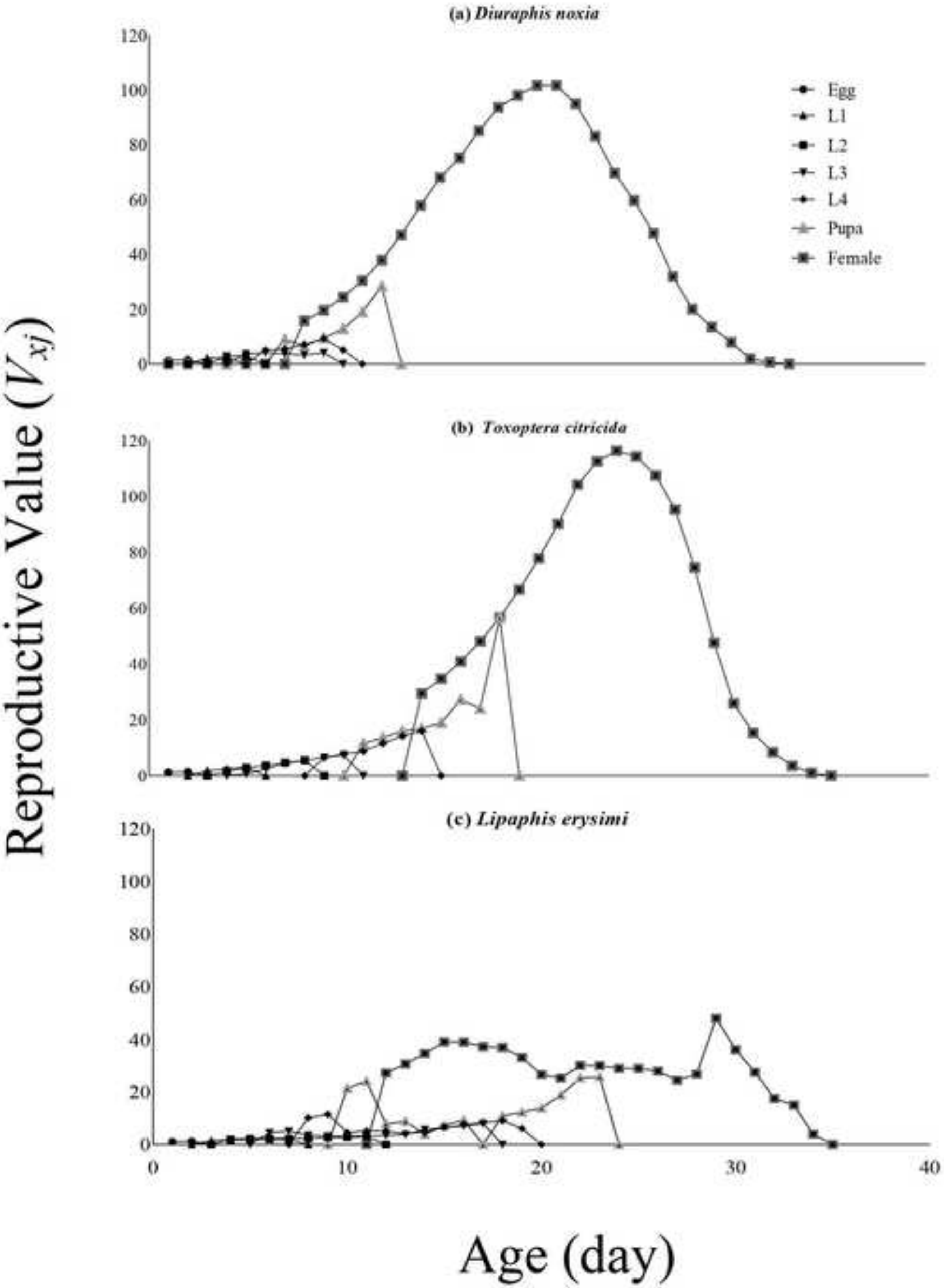

 Click here to access/download;Figure;Life expectency Fig 4.tiff

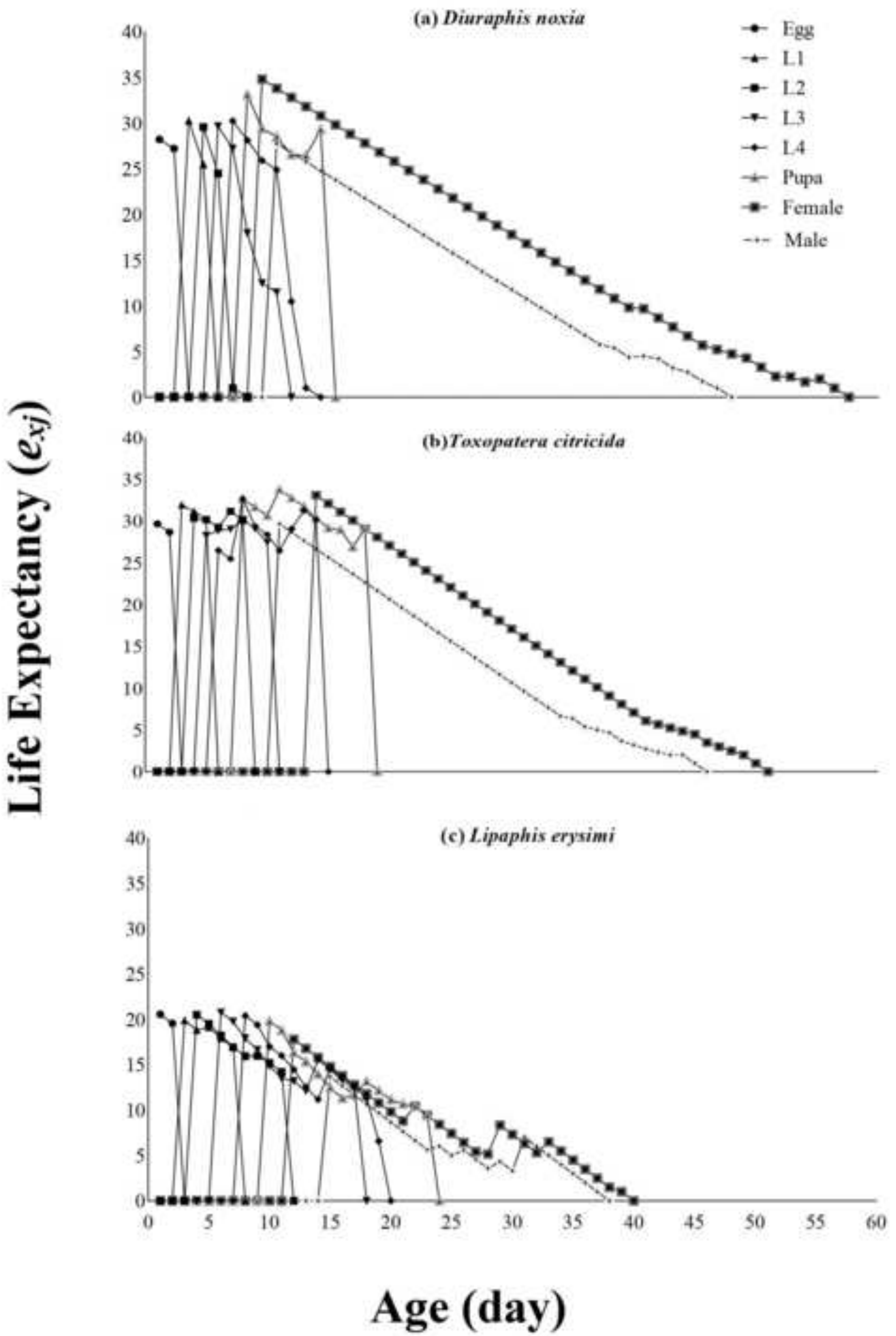

Figure 5

Click here to access/download;Figure;Larvae Fig 5.tiff

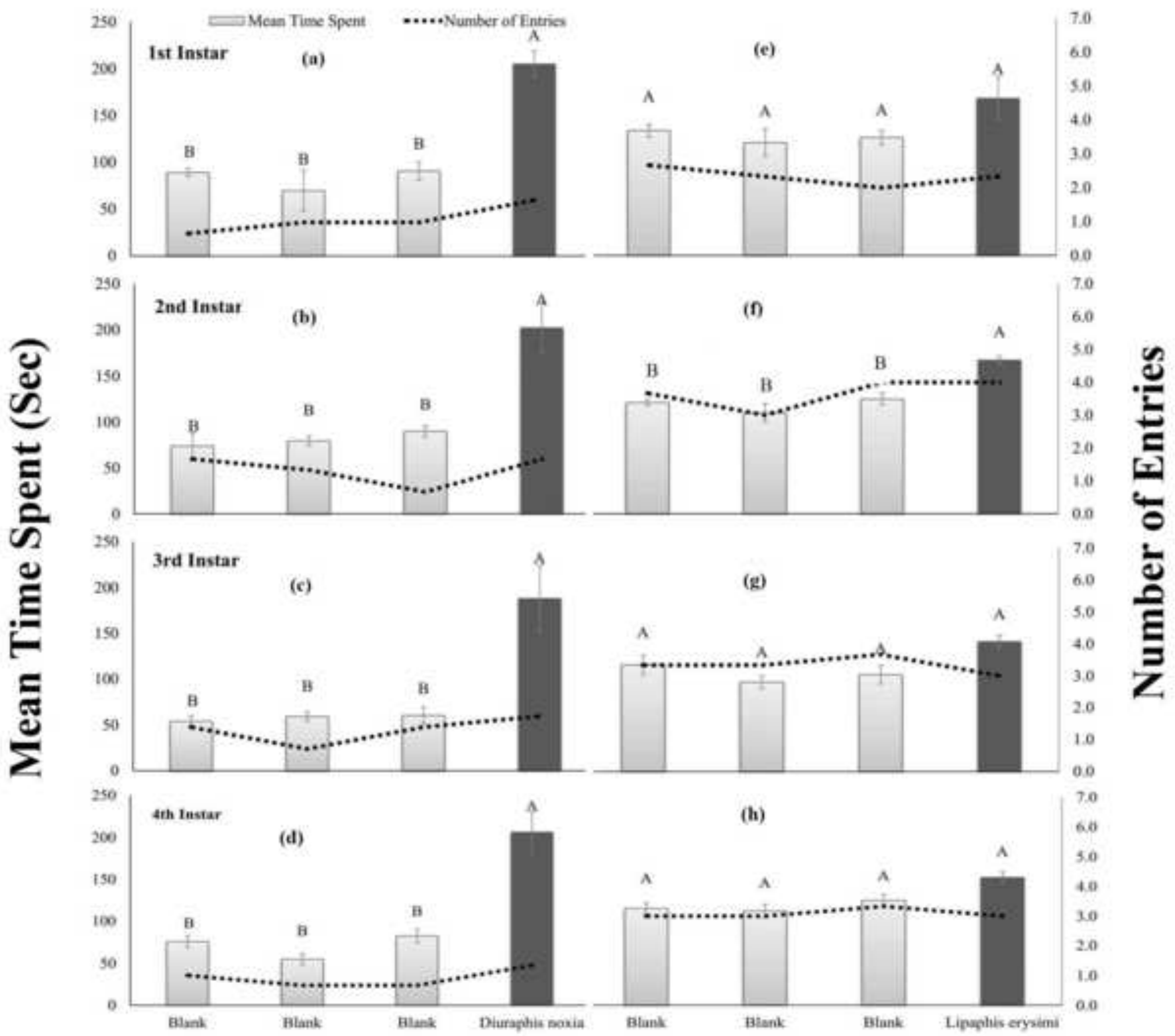

Figure 6

Click here to access/download;Figure;Matures Fig 6.tiff

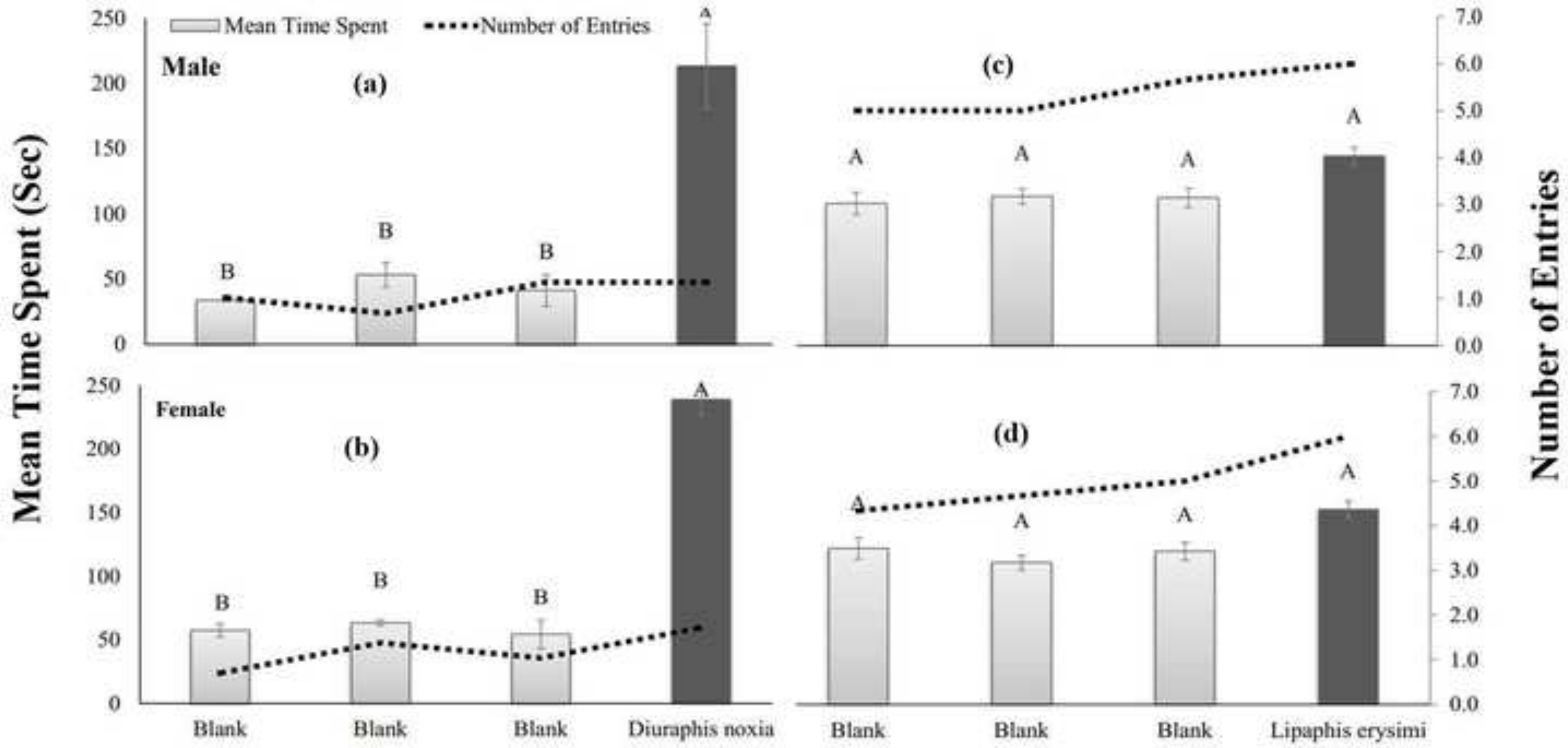

Supplement: Attachment — Submitted filename: PONE-D-22-16646_Boopathi.pdf [file pone.0289682.s002.pdf]
